# PlMYB308 Regulates Flower Senescence by Modulating Ethylene Biosynthesis in Herbaceous Peony

**DOI:** 10.3389/fpls.2022.872442

**Published:** 2022-05-31

**Authors:** Xiaotong Ji, Meiling Wang, Zhuangzhuang Xu, Kai Wang, Daoyang Sun, Lixin Niu

**Affiliations:** College of Landscape Architecture and Arts, Northwest A&F University, Yangling, China

**Keywords:** herbaceous peony, flower senescence, PlMYB308, ethylene, plant hormones

## Abstract

Herbaceous peony is an important cut-flower plant cultivated worldwide, but its short vase life substantially restricts its economic value. It is well established that endogenous hormones regulate the senescence process, but their molecular mechanism in flower senescence remains unclear. Here, we isolated a MYB transcription factor gene, *PlMYB308*, from herbaceous peony flowers, based on transcriptome data. Quantitative real-time PCR analysis showed that *PlMYB308* is strongly up-regulated in senescing petals, and its expression was induced by abscisic acid or ethylene and reduced by gibberellin in petals. Treatment with abscisic acid or ethylene accelerated herbaceous peony petal senescence, and gibberellin delayed the process. *PlMYB308* silencing delayed peony flower senescence and dramatically increased gibberellin, but reduced ethylene and abscisic acid levels in petals. *PlMYB308* ectopic overexpression in tobacco accelerated flower senescence and reduced gibberellin, but increased ethylene and abscisic acid accumulation. Correspondingly, five endogenous hormone biosynthetic genes showed variable expression levels in petals after *PlMYB308* silencing or overexpression. A dual-luciferase assay and yeast one-hybrid analysis showed that PlMYB308 specifically binds the *PlACO1* promoter. Moreover, treatment with ethylene and 1-MCP can accelerate *PlMYB308* silencing-reduced senescence and delay *PlMYB308*- overexpression-induced senescence. We also found that *PlACO1* silencing delayed senescence in herbaceous peony petals. Taken together, our results suggest that the PlMYB308-*PlACO1* regulatory checkpoints positively mediate the production of ethylene, and thus contribute to senescence in herbaceous peony flowers.

## Introduction

Senescence is a complex coordinated process at the whole-organ, tissue, and cellular levels (Tripathi and Tuteja, 2014). In agricultural production, particularly in transportation and storage, senescence dramatically decreases crop yield and causes economic loss in commercial crops and ornamental plants (In et al., 2016). Nowadays, physiological, biochemical, and molecular approaches have been used to study the senescence process in various plant organs, such as fruits (Kou et al., 2012), leaves (Zhang and Gan, 2012), and flowers (Chen et al., 2011). Senescence phenotypic changes, such as color fading or wilting, are mediated by complex pathways, and the degradation of polymers or nucleic acids and protein biosynthesis can influence these pathways. Thousands of up- or down-regulated structural genes and transcription factors (TFs) have been reported to be implicated in plant senescence (Guo and Gan, 2012; Hong et al., 2013; Yamazaki et al., 2020).

Abiotic and biotic stresses may trigger flower senescence. Numerous studies have provided evidence that there is an intricate and complex network among phytohormones (Zhang and Zhou, 2013), and it has been well known that ethylene is an important endogenous hormone regulating flower senescence. A burst of ethylene production can cause flower senescence in many plants, such as petunia and carnation (Saks and Staden, 1993; Onoue et al., 2000; Chang et al., 2003). Treating *Tropaeolum majus* with exogenous ethylene accelerates its flower senescence while treatment with ethylene biosynthesis inhibitors delays the process (Silva et al., 2015). The mechanisms of ethylene function in flower opening and senescence have been widely studied. In rose, *RhETR3* promotes flower opening as an important receptor gene in the mediation of ethylene signaling (Ma et al., 2008). In *Arabidopsis*, the repression of microRNA164 expression by ethylene promotes flower senescence (Li et al., 2013). In *Helianthus annuus*, *HaHB-4* up-regulation in response to ethylene delays the senescence process (Manavella et al., 2006). Besides ethylene, other phytohormones, such as gibberellin (GA) and abscisic acid (ABA), are involved in flower senescence. GA can delay senescence. In daffodil, GA_3_ treatment increased the cut flower vase life (Hunter et al., 2002, 2004). In *Glandularia hybrida*, GA_3_ can increase cell size and promote the expansion of petals (Li et al., 2015), while in *Cucumis sativus*, GA_4_ can regulate organ growth (Lange and Lange, 2016). ABA is a hormone that regulates flower senescence (Panavas et al., 1998). In cut rose, low ABA levels were detected during early flower development, peaked during flower development, and finally decreased during flower senescence (Kumar et al., 2008). In lily, ABA application accelerated petal senescence. In peony, ethylene, ABA, and GA also play crucial roles during flower senescence. Shi et al. (2008) found that GA content gradually decreased during flower senescence. Wang and Dong (2009) found that ‘Luo Yang Hong’ flowers treated with ABA could open earlier and had longer best viewing periods. They inferred that ABA may influence ‘Luo Yang Hong’ flower senescence by increasing ethylene production (Wang and Dong, 2009). Jiang et al. (2012) also found that ethylene and ABA are two important phytohormones regulating peony cut flower senescence.

In addition to their individual effect, interactions between ethylene and GA or ethylene and ABA have also been reported to play crucial roles in flower senescence. The interaction between ethylene and GA modulates *DELLA* gene expression and regulates petal expansion (Luo et al., 2013). In petunia, ethylene and ABA play an important role in the initiation of flower senescence. *PhHD-Zip* mediates the interaction between ethylene and ABA, positively regulating ABA production and promoting flower senescence (Chang et al., 2014). Although many researchers have attempted to understand the senescence-modulating network in flowers, the cross-talk between plant hormones and the complex signaling pathways mediating flower senescence are still unclear.

MYB proteins comprise a large and functionally diverse TF family in all eukaryotes (Liu et al., 2019). They have highly conserved MYB domains binding to specific DNAs. In *Arabidopsis*, MYB proteins function as critical mediators of many different biological activities, suggesting their extensive functional diversification. In the past few decades, many members of the MYB family have been characterized as being involved in various biological processes, including plant development, cell shaping, hormone signaling, and biotic or abiotic stress tolerance (Shan et al., 2012). For instance, *Arabidopsis AtMYB105* and *AtMYB117* control lateral organ separation and axillary meristem formation upstream of *AtMYB37* (Lee et al., 2009). *MYB96* transcriptional activation contributes to plant tolerance to drought stress by mediating ABA signaling and cuticular wax biosynthesis in *Arabidopsis* (Seo et al., 2011). *AtMYB30* has also been shown to act in the brassinosteroid pathway controlling hypocotyls cell elongation in seedlings (Li et al., 2010). The R2R3 MYB TF is a key component of the MBW complex which affects anthocyanin accumulation (Stracke et al., 2001; Li et al., 2020). However, despite these observations, the modes of action of MYB308 TFs in hormone-regulated flower senescence of herbaceous peony remain unclear.

The commodity value of herbaceous peony flowers largely depends on an excellent flowering quality and long vase life (Yang et al., 2020). Thus, the regulatory mechanism of flower senescence is an important topics in the postharvest physiological research of herbaceous peony. Here, we report that an MYB family member, PlMYB308, participates in ethylene accumulation in herbaceous peony petals. PlMYB308 silencing in herbaceous peony and overexpression in tobacco increased and decreased flower longevity, respectively, highlighting its crucial role in flower senescence regulation.

## Materials and Methods

### Plant Materials and Growth Conditions

To avoid variation in gene expression levels in multi-layered petals, a herbaceous peony cultivar (*Paeonia lactiflora* ‘hangshao’) with single-layered petals was used in this study. Plants were grown in the germplasm resource garden of Northwest Agriculture and Forestry University. The clean floral samples were collected at the first day after flowering. They were immediately placed into tap water, and transferred to the laboratory within 1 h. The samples were kept in deionized water for the VIGS assay to silence *PlMYB308* (Ma et al., 2005). The petals used for expression assessment were collected at four developmental stages: the first day after flowering (D1); the second day after flowering (D2); the fourth day after flowering (D4); the sixth day after flowering (D6). All samples were immediately frozen in liquid nitrogen and stored at −80°C (Du et al., 2015). The seeds of tobacco (*Nicotiana tabacum*) were germinated in a growth chamber at 24/20°C day/night temperature, with a 70% relative humidity and 16/8 h light/dark cycle (Ji et al., 2021). These plants were used for ectopic overexpression experiment of *PlMYB308*.

### Hormone Treatments

In the experiment to detect the expression level of *PlMYB308*, 2 days after anthesis, collected herbaceous peony flowers for hormone treatments. Flowers with stems (∼25 cm) were placed in a vase containing 100 μM GA_3_, 200 μM ethrel, 100 μM ABA. Mock samples were placed in a vase containing distilled water without any phytohormones. Flowers were collected at 0, 24, 36, and 48 h after treatment. For each treatment, three individual flowers were used.

In the experiment to observe the effect of hormone on flower senescence, collected herbaceous peony flowers at the first day after flowering. Flowers with stems (∼25 cm) were placed in a vase containing 100 μM GA_3_, 200 μM ethrel, 100 μM ABA, 100 μM GA_3_ + 200 μM ethrel, 100 μM GA_3_ + 100 μM ABA. Mock samples were placed in a vase containing distilled water without any phytohormones. Recorded and photographed at the first day, second day, fourth day, sixth day, and eighth day after treatment. For each treatment, three individual flowers were used.

### Identification of *PlMYB308*

We use TIANGEN RNA Prep Pure Plant kit (Tiangen, Beijing, China) to isolated the total RNA of herbaceous peony petals. The first-strand cDNA was synthesized using a PrimeScript^®^ RT reagent Kit (Takara, Otsu, Shiga, Japan). The *PlMYB308* gene was PCR-amplified using specific primers (Supplementary Table 6). Through a BLAST search against non-redundant GenBank databases in the NCBI, the highly homologous proteins to the deduced polypeptides encoded by the *PlMYB308* gene were obtained. We used DNAMAN software (version 8.0) to compare the homologous sequence of PlMYB308 in herbaceous peony and other species and used the Neighbor-Joining method (NJ) of MEGA software (version 7.0) to construct a phylogenetic tree. The conserved domain was identified by SMART^1^.

### Subcellular Localization

For subcellular localization analysis, the *PlMYB308* ORF region without the stop codon was inserted into the binary vector pCAMBIA1301-GFP to generate 35S::*PlMYB308*-GFP fusion construct. Injected this constructed plasmid to the abaxial side of tobacco leaves. After incubation at 25°C for 16 h in the dark, we observed samples under a confocal laser scanning microscope.

### Virus-Induced Gene Silencing Assay

The silencing of *PlMYB308* and *PlACO1* by tobacco rattle virus (TRV)-based VIGS was performed as previously described (Dai et al., 2012). A 199-bp fragment of the *PlMYB308* region and a 216-bp fragment of *PlACO1* were introduced into the TRV vector to generate the TRV-*PlMYB308* and TRV-*PlACO1* constructs, which were used to specifically silence *PlMYB308* and *PlACO1* in herbaceous peony. We electrotransformed TRV1, TRV-*PlMYB308*, and TRV-*PlACO1* plasmids to GV3101 and cultured the transformed bacteria in LB media with 40 mg l^–1^ kanamycin, 20 mg l^–1^ gentamicin, 10 mM MES, and 20 μM acetosyringone. We centrifugated and harvested the cells, resuspended them to an OD600 of 4.0, and used the inoculation buffer with 10 mM MgCl_2_, 10 mM MES, and 200 μM acetosyringone. We mixed TRV1 and TRV-*PlMYB308* plasmids, or TRV1 and TRV-*PlACO1* plasmids in a 1:1 ratio, and then incubated them at room temperature for 3 h. Using a hole punch, we excised 1-cm diameter disks from the center of petals of herbaceous peony flowers obtained on the first day after flowering. We immersed petal disks in the bacterial suspension solution and infiltrated them under a vacuum at 0.9 MPa for 15 min. We then released the vacuum and washed the petal disks in deionized water, kept them in deionized water for 1 day at 8°C, and then kept them at 23°C. Samples for RNA isolation were collected on the first (D1), second (D2), fourth (D4), and sixth (D6) day after treatment. Twenty petal disks were randomly selected and mixed as a biological replicate. Three biological replicates were used for each measurement.

### Stable Transformation of *Nicotiana tabacum*

The transformation and regeneration of *Nicotiana tabacum* plants were performed using the leaf disk method. At least 10 independent transgenic lines were generated. Homozygous lines from T2 generations were used for the follow-up experiments.

### Quantitative Real-Time PCR Assay

A quantitative real-time PCR (qRT-PCR) assay was used to determine the expression levels of *PlMYB308* and other flower senescence-associated genes in herbaceous peony and tobacco plants. The sequences of related genes were obtained from the NCBI GenBank database^2^. We designed specific primers according to the cDNA sequences of all genes tested (Supplementary Table 6). We used *PlUB* and *NtEF-1α* as internal controls to normalize expression data in herbaceous peony and tobacco, respectively. At the end of the qRT-PCR program, the melting curve program was used to ensure the specific amplification. We used the 2^–ΔΔCT^ comparative threshold cycle (Ct) method to calculate the relative gene expression levels. Three biological replicates were used for each measurement.

### Decay Rates and Flower Longevity

The decay rates of the herbaceous peony petal disks were recorded per day after inoculation. Flower longevity was recorded from anthesis until complete wilting of corollas. Three hundred *PlMYB308* silenced petal disks and 300 TRV empty vector control petal disks from the VIGS assay of herbaceous peony were used for the detection of decay rates. Ten flowers from each *PlMYB308*-overexpressing line and 10 flowers from WT plants in the ectopic overexpression assay of tobacco plants were used for longevity detection.

### Anthocyanin Content Measurement

Total anthocyanin levels in herbaceous peony petal disks and *Nicotiana tabacum* corollas were determined according to a previously described method (Luo et al., 2017). For herbaceous peony petal disks, 20 petal disks were randomly selected and mixed as a biological replicate. For *Nicotiana tabacum*, three flowers were randomly selected and mixed as a biological replicate. Three biological replicates were analyzed for each sample.

### Measurement of Ethylene, Gibberellin, and Abscisic Acid Production

Ethylene production was measured at D4 after infiltration with a TRV empty vector control and TRV-*PlMYB308* in petal disks of herbaceous peony, and at D5 in WT and *PlMYB308*-overexpressing tobacco plants. About 100 mg of samples were placed into airtight tubes at 26°C for 4 h. Approximately 1 ml of gaseous samples were collected using a gastight hypodermic syringe and injected into a gas chromatographer (GC-8A; Shimadzu, Kyoto, Japan) for ethylene detection. GA and ABA productions were measured as previously described (Pan et al., 2010). Three biological replicates were analyzed for each sample.

### Dual-Luciferase Transient Transfection Assay

The pGreenII 62-SK construct including the coding region of *PlMYB308*, and the pGreenII 0800 constructs containing different promoter sequences were prepared (Supplementary Figure 4). The experimental steps were as previously described (Zong et al., 2016).

### Yeast One-Hybrid Analysis

The *PlACO1*, *PlACO3*, and *PlACS1* promoters were cloned into the pHIS2 plasmid. PlMYB308 was cloned into the pGAD-T7Rec plasmid. All constructs were transformed into the yeast strain Y187. Yeast was grown in SD-Ura-Leu-His medium and then spotted onto SD-Ura-Leu-His medium with different concentrations of 3-aminotriazole (Sigma) in different dilutions. The screening conditions of *PlACO1*, *PlACO3*, and *PlACS1* promoters in pHIS2 were 100 mM 3-aminotriazole, 30 mM 3-aminotriazole, and 10 mM 3-aminotriazole, respectively.

### Statistical Analysis

In this study, all experiments were carried out with a minimum of three biological replicates for different individual plants. The significance of difference was determined through Student’s *t-*test at *P* value < 0.05.

## Results

### GA_3_, Abscisic Acid, and Ethylene Regulate Herbaceous Peony Flower Senescence

We assessed the visible flower senescence of herbaceous peony upon the application of GA_3_, ethylene, ABA, GA_3_ + ethylene, and GA_3_ + ABA (Supplementary Figure 1). As shown in the figure, the control flower began to wilt at 6 days after treatment. In ABA- or ethylene-treated flowers, flower senescence began at 4 days after treatment. Compared with untreated controls, GA_3_ treatment alone clearly delayed senescence, and flowers treated with a combination of GA_3_ + ABA or GA_3_ + ethylene showed a longer vase life than those treated with ABA or ethylene alone. Taken together, these results indicate that GA_3_ delays flower senescence, ABA and ethylene accelerates flower senescence, and GA_3_ plays an antagonistic role in ABA- and ethylene-induced petal senescence.

### *PlMYB308* Expression Changes During Flower Senescence and After Abscisic Acid, Ethylene, and GA_3_ Treatments in Herbaceous Peony Flowers

**FIGURE 1:**
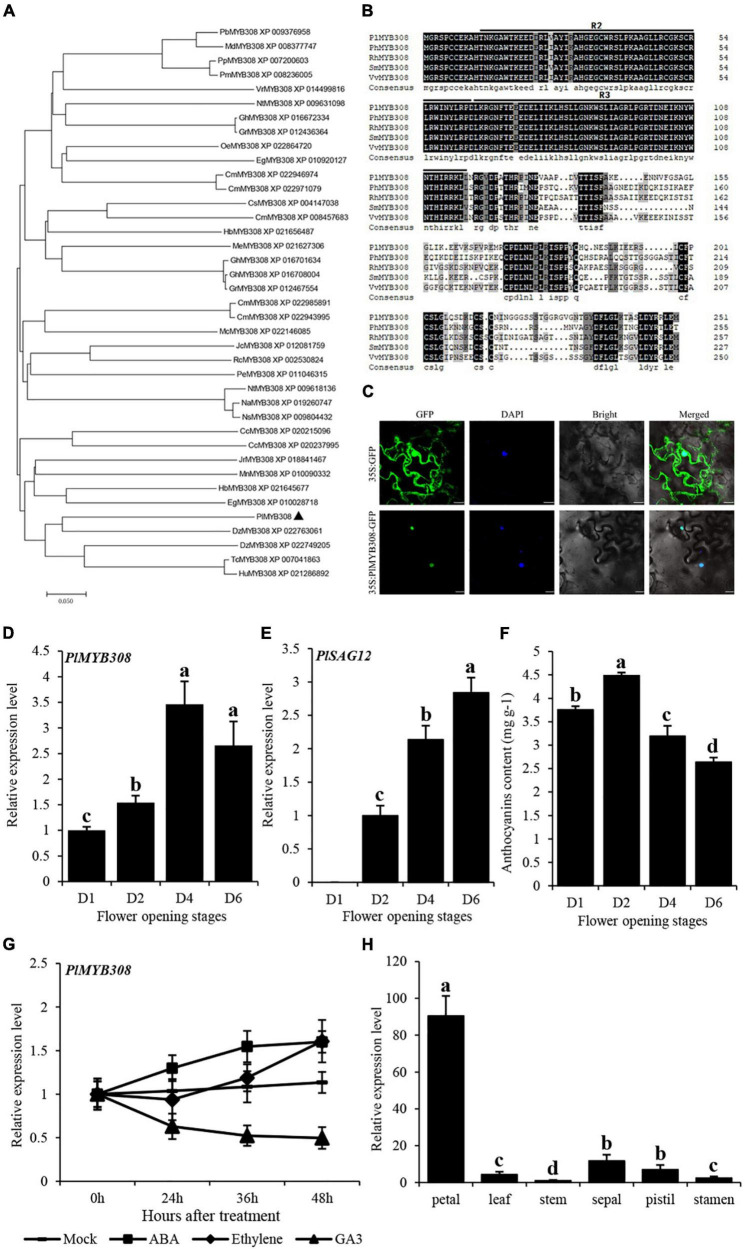
Phylogenetic analysis, homologous sequences, subcellular location, and expression levels of *PlMYB308*. **(A)** Phylogenetic tree generated using various MYB308 protein sequences from *Eucalyptus grandis, Ricinus communis, Fragaria vesca* L. and other plant species. Bootstrap values indicated the divergence of each branch, the scale indicated branch length. **(B)** Homologous sequences compared using various MYB308 protein sequences from *Paeonia lactiflora, Petunia hybrida, Salvia miltiorrhiza*, and *Vitis vinifera*. **(C)** Subcellular localization of GFP fusion of PlMYB308. Scale bars = 20 μm. **(D,E)** Quantitative RT–PCR analysis of PlMYB308 and PlSAG12 expressions in herbaceous peony petals at various opening stages. PlUB was used as an internal control. **(F)** The anthocyanin content at various flower opening stages were determined. **(G)** Expression levels of *PlMYB308* under hormones treatment. Herbaceous peony flowers harvested at anthesis then placed in tubes with water (mock), 0.1 mM ABA, 0.2 mM ethephon, and 50 μM GA3. **(H)** Relative expression levels of *PlMYB308* in different tissues. Error bars showed SD of the means of three biological replicates. a, b, c, and d indicated significant difference at *p* ≤ 0.05 level by Duncan test. Error bars show SD of the means of three biological replicates.

The full-length cDNA sequence of *PlMYB308*, containing a 759-bp open reading frame (ORF) region, was isolated from the floral tissues of herbaceous peony. Amino acid alignments and phylogenetic analyses showed that PlMYB308 was relatively close to the MYB308s from other plant species (Figure 1A). All homologous sequences exhibited high amino acid conservation in the R2R3-MYB domains. Beyond these domains, the amino acid sequences diverged greatly for different MYB308 homologs (Figure 1B). Subcellular localization showed that PlMYB308 was localized in the cell nucleus (Figure 1C).

The complete flower senescence process of herbaceous peony is shown in Supplementary Figure 2. qRT-PCR data showed that the transcript abundance of *PlMYB308* significantly increased during flower senescence and reached the highest level at 4 days after anthesis, decreasing after it (Figure 1D). This process was accompanied by an increase in the transcript levels of the senescence-related gene *PlSAG12* and a decrease in anthocyanin accumulation levels (Figures 1E,F). This implied that *PlMYB308* likely plays an essential role in petal senescence. We then determined *PlMYB308* expression levels in herbaceous peony petals after different hormone treatments. Flowers treated with ABA or ethylene displayed higher *PlMYB308* expression than untreated controls. Flowers treated with GA_3_ showed the opposite tendency (Figure 1G). To know if *PlMYB308* is petal-specific, we analyzed *PlMYB308* transcripts in petal, leaf, stem, sepal, pistil, and stamen. We found that *PlMYB308* was expressed in all flower organs, with the highest abundance in petals (Figure 1H).

### *PlMYB308* Silencing and Overexpression Influence Flower Longevity

To uncover the function of *PlMYB308* in flower senescence, we silenced it in herbaceous peony using a VIGS approach and overexpressed it in tobacco plants using a stable genetic transformation technique.

To find an easily manipulated and replicated experimental system to study herbaceous peony flower senescence, we investigated whether it was possible to use petal disks instead of the whole herbaceous peony flower. We compared some senescence-related markers such as soluble protein content, soluble sugar content, MAD content, anthocyanin content, and expression level of *PlSAG12*. All these markers showed the same trends in petal disks and whole flowers, and we concluded that petal disks provide an appropriate experimental system (Supplementary Figure 3).

**FIGURE 2:**
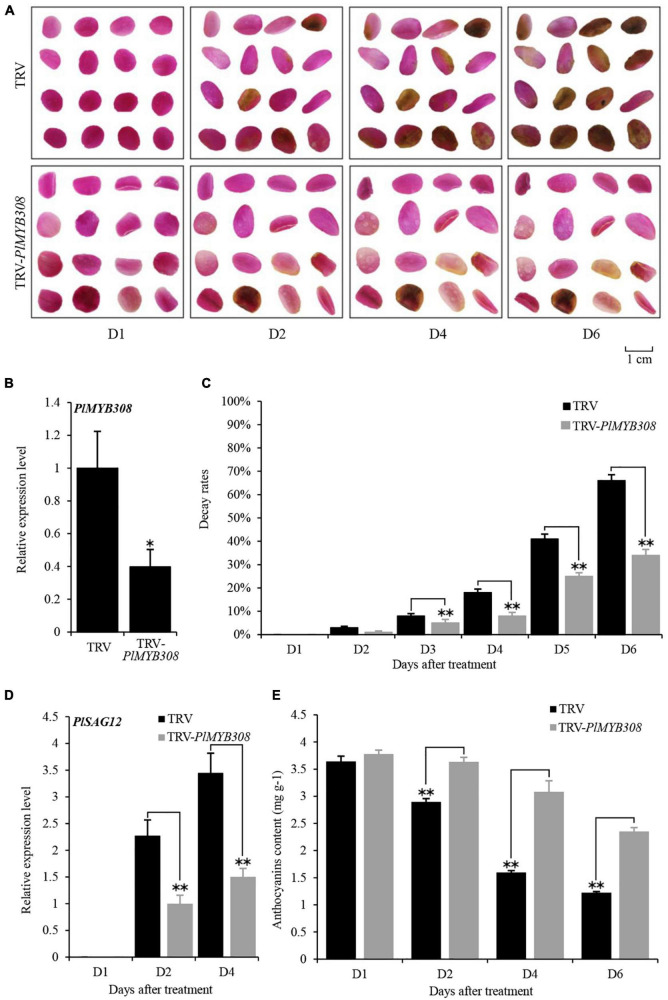
*PlMYB308* silencing delayed senescence of herbaceous peony petal disks. **(A)** The phenotypes of the petal disks were photographed at D1, D2, D4, D6 after infiltration with TRV empty vector and TRV-*PlMYB308*. **(B)** Abundances of *PlMYB308* transcript in empty vector- and TRV-*PlMYB308*-infected petal disks at D4. PlUB was used as an internal control. **(C)** Decay rates of the petal disks infiltrated with TRV empty vector and TRV-*PlMYB308*. Means ± SD for 300 petal disks. **(D)** Expression levels of *PlSAG12* by quantitative real-time PCR in empty vector- and TRV-*PlMYB308*-infiltrated petal disks. **(E)** The anthocyanin content at D1, D2, D4, D6 after infiltration with TRV empty vector and TRV-*PlMYB308*. Error bars showed SD of the means of three biological replicates. Asterisks indicated statistically significant differences by Student’s *t*-test (*P < 0.05, **P < 0.01).

A 199-bp fragment of the *PlMYB308*-specific 3′ end region was introduced into a TRV vector to generate the TRV-*PlMYB308* construct, which was used to specifically silence *PlMYB308* in herbaceous peony petal disks. In the VIGS assay, the phenotype of petal color fading started at D2 after infiltration with empty vector, and petal disks almost turned brown at D6. In contrast, the *PlMYB308*-silenced disks exhibited a delayed senescence phenotype, with only slight color fading at D6 after inoculation (Figure 2A). The transcript abundance of *PlMYB308* was remarkably reduced in TRV-*PlMYB308-*infiltrated petal disks, compared with those infiltrated with TRV empty vector controls (Figure 2B). Approximately 3% of the petal disks began to decay at D2, and the decay rate reached 66% at D6 in the controls. However, in *PlMYB308*-silenced disks, relatively lower petal decay rates were observed at D2 (1%) and D6 (32%) after inoculation (Figure 2C). Moreover, the expression levels of *PlSAG12* in *PlMYB308*-silenced petal disks were significantly lower than those in the empty vector controls (Figure 2D). Anthocyanin content in *PlMYB308*-silenced petal disks was significantly higher than that in TRV controls (Figure 2E). These results indicated that senescence progress is delayed by *PlMYB308* silencing.

**FIGURE 3:**
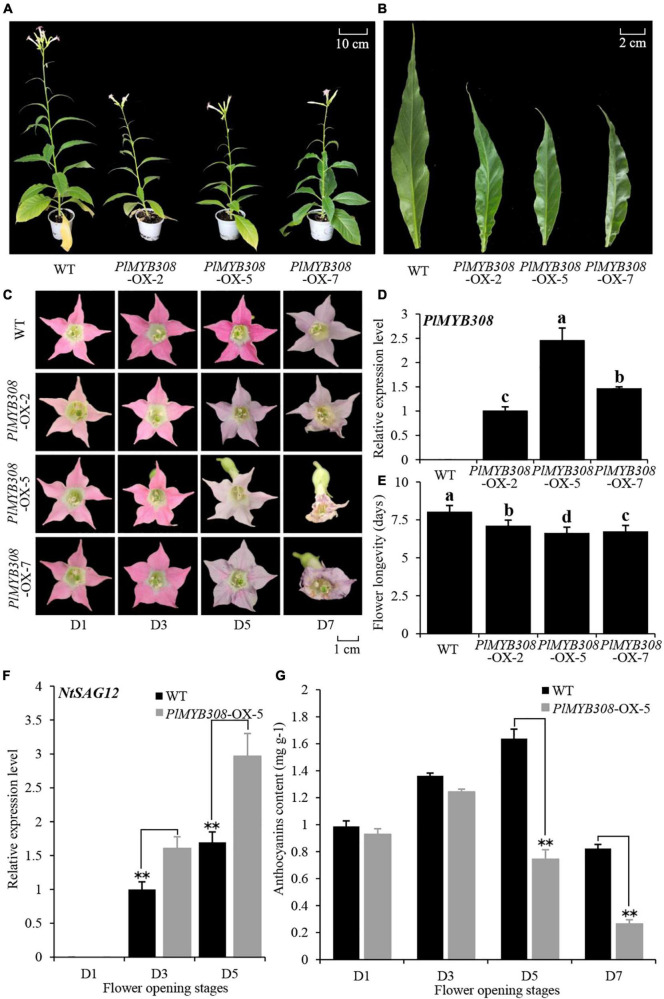
*PlMYB308* overexpression accelerated flower senescence in tobacco. **(A,B)** *PlMYB308* overexpression led to shorter stems and smaller leaves than the wild-type (WT) control. Ten-week-old plants of WT and PlMYB308-overexpressing lines (OX-2, OX-5, and OX-7) were recorded and photographed. The fully expanded leaves were collected from the second leaves from the top of the branch. **(C)** The floral phenotypes were photographed at D1, D3, D5 and D7 after anthesis. **(D)** Expression levels of PlMYB308 in wild-type (WT) plants and *PlMYB308*-overexpressing transgenic plants (*PlMYB308*-OX) by quantitative real-time PCR at D1. *PlMYB308*-OX-2, *PlMYB308*-OX-5 and *PlMYB308*-OX-7, different lines of PlMYB308 overexpression. **(E)** Longevities of attached flowers in WT and *PlMYB308*- overexpressing transgenic tobacco plants. Means ± SD for 10 flowers. **(F)** Expression levels of NtSAG12 in WT and PlMYB308-overexpressing line (OX-5) at given time points. **(G)** The anthocyanin content at D1, D3, D5, D7 after anthesis. Error bars show SD of the means of three biological replicates. Asterisks indicated statistically significant differences (*P < 0.05, **P < 0.01, Student’s *t*-test).

We transformed the model plant tobacco with a full-length coding sequence of *PlMYB308* cDNA driven by the constitutive Lac 35S promoter. Transgenic tobacco plants displayed visibly shorter stems and smaller leaves than the wild-type (WT) plants (Figures 3A,B). Different transgenic lines (# 2, # 5, and # 7) showed accelerated flower senescence compared to that in WT plants (Figures 3C,E). A strong constitutive expression of *PlMYB308* was found in three transgenic lines (Figure 3D). The line (# 5) exhibiting the maximum expression of *PlMYB308* was selected for further analysis. *NtSAG12* transcript abundance in *PlMYB308-*overexpressing transgenic plants was significantly higher than that in WT plants during flower senescence (Figure 3F). Anthocyanin content was significantly lower in *PlMYB308-*overexpressing transgenic plants compared to that in WT plants 5 days after anthesis (Figure 3G).

### *PlMYB308* Affects Ethylene, Abscisic Acid, and Gibberellin Production and Modulates the Expression of Some Hormone Biosynthetic Genes

**FIGURE 4:**
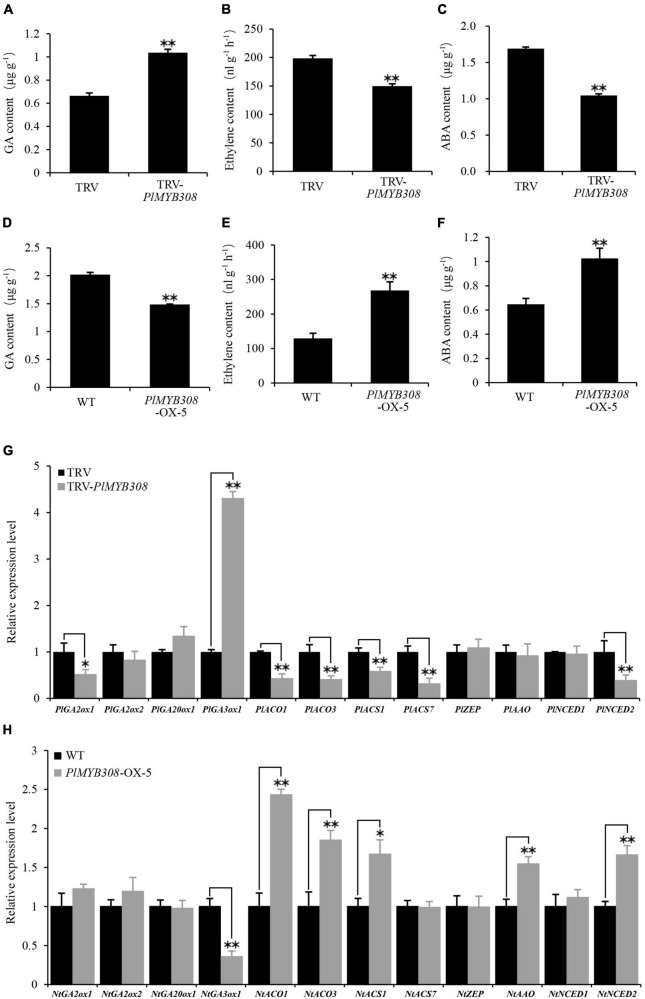
Effect of *PlMYB308* overexpression and silencing on GA, ethylene, and ABA production and hormone biosynthesis-related genes expression levels. **(A–C)** Content of GA, ethylene, and ABA in TRV empty vector- and TRV-*PlMYB308*-infiltrated herbaceous peony petal disks. GA, ethylene, and ABA production was measured at D4 after infiltration. **(D–F)** Content of GA, ethylene and ABA in wild-type and *PlMYB308*-overexpressing transgenic tobacco line (OX-5). GA, ethylene, and ABA production was measured at D5 after anthesis. **(G)** The petal disks were sampled for gene expression analysis at D4 after infiltration with TRV empty vector and TRV-*PlMYB308*. *PlUB* was used as an internal control. Relative expression levels were normalized to the empty vector control. **(H)** The tobacco flowers were sampled for gene expressions analysis at D5 after anthesis. NtEF-1α was used as an internal control. Relative expression levels were normalized to the WT. Error bars show SD of the means of three biological replicates. Asterisks indicate statistically significant differences (*P < 0.05, **P < 0.01, Student’s *t*-test).

As ethylene, ABA, and GA are important hormones regulating flower senescence, we investigated their accumulation in *PlMYB308-*silenced herbaceous peony petal disks and *PlMYB308-*overexpressing transgenic tobacco plants. GA production significantly increased in *PlMYB308*-silenced petal disks at D4 after infiltration compared with the TRV empty vector controls (Figure 4A). On the contrary, GA levels in *PlMYB308*-overexpressing flowers at D5 after anthesis were much lower than those in WT flowers (Figure 4D). Ethylene accumulation markedly decreased in petal disks upon *PlMYB308* silencing but increased in transgenic tobacco lines upon *PlMYB308* overexpression, compared to the controls (Figures 4B,E). ABA contents showed the same trends as those of ethylene in *PlMYB308*-silenced or -overexpressing plants (Figures 4C,F). These results suggested that *PlMYB308* regulates flower senescence by mediating the ethylene, ABA, and GA biosynthesis.

To further explore the role of *PlMYB308* in hormone accumulation regulation, we examined transcript levels of 12 different genes from herbaceous peony and tobacco plants related to hormone biosynthesis. Compared with controls, *PlGA3ox1* was significantly up-regulated in *PlMYB308*-silenced petal disks, while *PlGA2ox1*, *PlACO1*, *PlACO3*, *PlACS1*, *PlACS7*, and *PlNCED2* were down-regulated (Figure 4G). Similarly, *NtGA3ox1* expression levels substantially decreased in *PlMYB308*-overexpressing transgenic tobacco lines compared to WT, whereas transcript abundances of *NtACO1*, *NtACO3*, *NtACS1*, *NtAAO*, and *NtNCED2* increased (Figure 4H). These gene expression results were in agreement with those obtained from hormone measurements above.

### PlMYB308 Binds to the Promoter of the Ethylene Biosynthetic Gene *PlACO1*

Based on the results described above, the promoters of ethylene, ABA, and GA biosynthetic genes *PlACO1*, *PlACO3*, *PlACS1*, *PlNCED2*, and *PlGA3ox1* were isolated as potential targets of PlMYB308 transcriptional activation. Their promoter sequences were submitted to the online website PlantCare for predictive analysis, revealing a diversity of *cis*-acting elements within the promoter region (Supplementary Tables 1– 5).

**FIGURE 5:**
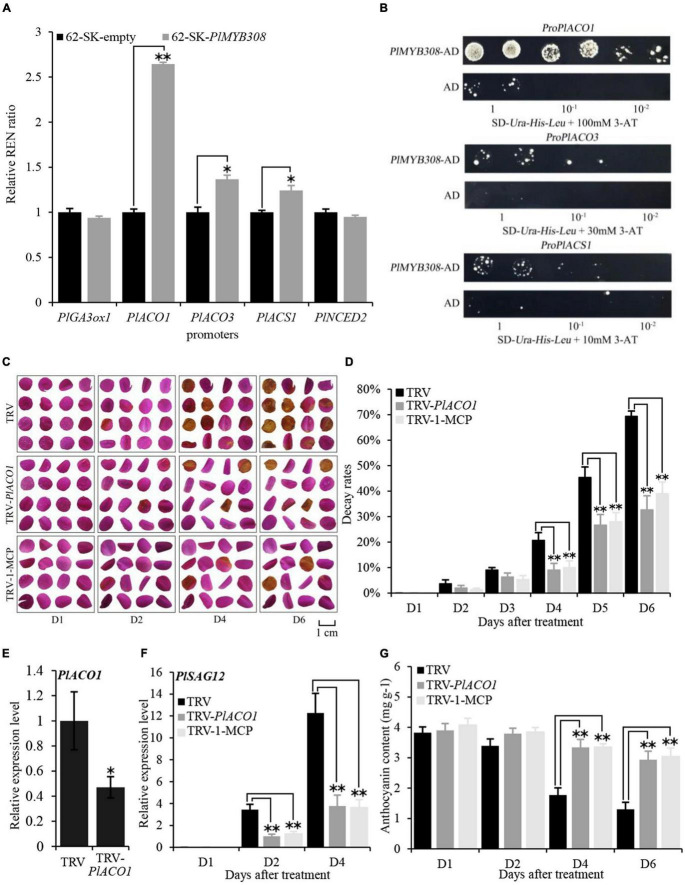
*PlMYB308* bound to the promoter of ethylene biosynthetic gene *PlACO1*, and *PlACO1* silencing delayed the senescence of herbaceous peony petals. **(A)** *PlMYB308* transactivated the promoter of *PlACO1*. The ratio of LUC/REN of the empty vector (62-SK) plus promoter was considered as a calibrator (set as 1). The activation is indicated by the ratio of LUC to REN. Data represent the mean ± SD of six independent repeats. **(B)** The yeast one-hybrid result of *PlMYB308*. The transformed yeast was grown on SD-Leu-His-Leu + 3-AT medium. The screening condition was 3-AT 100 mM, 3-AT 30 mM, and 3-AT 10 mM, from the top down. Cells were grown in liquid medium to an optical density at 600 nm of 1.0. The numbers 1,10-1,10-2 indicate the dilutions. **(C)** The phenotypes of the petal disks were photographed at D1, D2, D4, D6 (TRV: petal disks infiltration with TRV empty vector; TRV-*PlACO1*: petal disks infiltration with TRV-*PlACO1*; TRV-1-MCP: petal disks infiltration with TRV empty vector and treated with 1-MCP). **(D)** Decay rates of the petal disks. Means ± SD for 300 petal disks. **(E)** Abundances of *PlACO1* transcript in empty vector- and TRV-*PlACO1*-infected petal disks at D4. *PlUB* was used as an internal control. **(F)** Expression levels of PlSAG12 by quantitative real-time PCR in different petal disks. **(G)** The anthocyanin content at D1, D2, D4, D6. Error bars showed SD of the means of three biological replicates. Asterisks indicated statistically significant differences by Student’s *t*-test (*P < 0.05, **P < 0.01).

To determine whether the genes associated with ethylene, ABA, and GA biosynthesis were targeted by PlMYB308, a dual-luciferase assay was carried out. The co-expression of pGreenII 62SK-*PlMYB308* with the pGreenII 0800-*PlACO1* promoter resulted in a 2.5-fold rise in firefly luciferase (LUC) activity, demonstrating that PlMYB308 significantly activated the promoter of *PlACO1* (Figure 5A). This data suggested that PlMYB308 may function as a specific regulator of ethylene biosynthesis by targeting *PlACO1*.

To further identify the interaction between *PlACO1*, *PlACO3*, and *PlACS1* promoters and PlMYB308, these three promoters were used as bait in the yeast one-hybrid system. When PlMYB308 was transformed together with the *PlACO1*, *PlACO3*, and *PlACS1* promoters into yeast, only *PlACO1* promoters grew well in the 100 mM 3-aminotriazole His^–^ SD medium (Figure 5B). These results indicated that PlMYB308 bound the promoter of *PlACO1* and activated its expression in yeast cells. Based on the dual-luciferase and yeast one-hybrid results, we demonstrate that PlMYB308 binds to the promoter of the ethylene biosynthetic gene *PlACO1*.

### *PlACO1* Silencing Delays Senescence in Herbaceous Peony Petals

*ACO1* is a key regulatory gene in the ethylene biosynthesis pathway. As ethylene treatment accelerates flower senescence, we speculated that altering the expression of *PlACO1* may have a similar effect. Indeed, we found that using VIGS to suppress *PlACO1* expression delayed senescence in *PlACO1*-silenced petal disks compared to that in TRV controls, as was seen with 200 μM 1-MCP treatments (Figures 5C,D). The transcript abundance of *PlACO1* was remarkably reduced in TRV-*PlACO1*-infiltrates petal discs (Figure 5E). *PlSAG12* transcript levels were significantly lower, and the anthocyanin contents were significantly higher in the *PlACO1*-silenced and 1-MCP-treated disks compared to those in TRV controls (Figures 5F,G). Collectively, these results suggest that *PlACO1* plays a role in accelerating herbaceous peony petal senescence.

### PlMYB308 Mediates Ethylene Biosynthesis During Herbaceous Peony Flower Senescence

**FIGURE 6:**
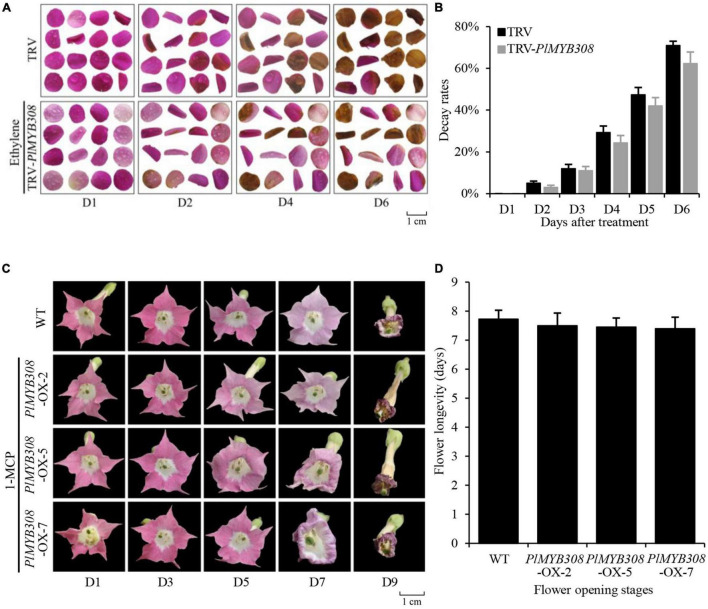
*PlMYB308* mediates ethylene biosynthesis during herbaceous peony flower senescence. **(A)** Treated PlMYB308-silenced petal disks with ethylene and photographed the phenotypes of the TRV empty vector and TRV-*PlMYB308* petal disks at D1, D2, D4, D6. **(B)** The decay rates of the petal disks were recorded from D1 to D6. Means ± SD for 200 petal disks (100 from TRV empty vector and 100 from TRV-*PlMYB308*-infected petal disks). **(C)** Treated *PlMYB308*-overexpressing transgenic flowers with 1-MCP and photographed the phenotypes of the WT flowers and *PlMYB308*-overexpressing transgenic flowers at D1, D3, D5, D7 and D9. **(D)** Longevities of the flowers were recorded. Means ± SD for 40 flowers (10 flowers from each *PlMYB308*-overexpressed lines and 10 flowers from WT plants).

As we had supposed, PlMYB308 may accelerate herbaceous peony flower senescence by inducing ethylene biosynthesis. To verify our hypothesis, we treated *PlMYB308*-silenced petal disks and *PlMYB308-*overexpressing transgenic flowers with 200 μM ethephon and 200 μM 1-MCP, respectively. When we treated *PlMYB308*-silenced petal disks with ethylene, color fading started on the second day both in *PlMYB308*-silenced petal disks and TRV control treatment. Disks had almost turned senescent at day 6 (Figure 6A). We recorded the decay rates of petal disks. In TRV controls, 5% of petal disks began to decay on the second day, and 71% turned to decay on the sixth day. *PlMYB308*-silenced disks showed a similar rate of senescence, 3% of petal disks began to decay on the second day, and 62% turned to decay on the sixth day (Figure 6B). This suggested that the senescence delay caused by *PlMYB308-*silencing can be restored by ethylene. We treated *PlMYB308*-overexpressing transgenic tobacco flowers with 1-MCP, and flowers from transgenic plants and WT plants had similar flower longevity (Figure 6C). We recorded the flower longevity, and that of intact WT flowers was 7.725 days and that of intact *PlMYB308*-overexpressing transgenic flowers was 7.4–7.5 days (Figure 6D), suggesting that 1-MCP can delay the overexpression of *PlMYB308*-induced senescence. Both results suggested that flower senescence regulated by *PlMYB308* could be influenced by ethylene content, further demonstrating that PlMYB308 mediates ethylene biosynthesis during herbaceous peony flower senescence.

## Discussion

**FIGURE 7:**
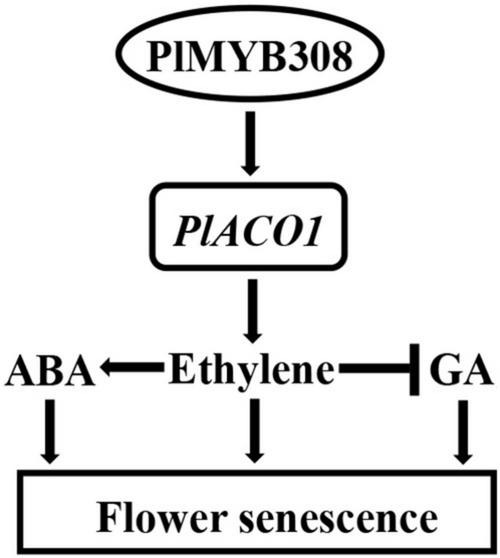
The model of *PlMYB308*-mediated regulation of flower senescence. Arrows indicate promotion, and leading dashes indicate inhibition.

Flower senescence is a complex biological process concurrent with the phenomenon of pigment fading. It is well established that plant hormones normally work in combination to play important roles in flower senescence. Here, we identified the MYB TF PlMYB308 in herbaceous peony, because of its sequence similarity to MYB308 in other plants, as being capable of binding to the promoter of an ethylene biosynthetic gene, *PlACO1*, and thereby specifically activating its expression. During flower senescence, the expression of *PlMYB308* increased, specifically activating *PlACO1* expression. A high expression of *PlACO1* increased ethylene content, and high ethylene content induced ABA biosynthesis and reduced GA biosynthesis. Taken together, the PlMYB308-*PlACO1* regulatory point mediates the interaction among ethylene, ABA, and GA networks during herbaceous peony flower senescence (Figure 7).

### PlMYB308 Is a Transcription Factor Associated With Herbaceous Peony Flower Senescence

Ethylene is an important plant hormone in herbaceous peony flower senescence. Exogenous ethylene accelerates flower senescence, while an inhibitor of ethylene extends this process (Si et al., 2021). However, as in other flowers, the senescence of herbaceous peony is modulated by diverse hormonal or environmental signals. These complex signals are regulated by thousands of transcription factors. In *Arabidopsis*, researchers have indicated that several transcription factors participate in flower senescence (Wagstaff et al., 2009), but the data do not suggest a role for the R2R3-MYB family. Based on our transcriptome data, PlMYB308 (an R2R3 MYB TF from herbaceous peony) was significantly up-regulated during flower senescence. In other plants, these TFs are usually associated with biotic and abiotic stresses. In *Arabidopsis*, an R2R3-MYB TF, *BOS1*, mediates responses to signals from both biotic and abiotic stress agents (Mengiste, 2003). In *Chrysanthemum*, *CmMYB2* mediates drought and salt tolerance improvement and flowering-time modulation (Hong et al., 2013). In *Populus trichocarpa*, an R2R3 MYB TF, *PtrSSR1*, regulates ABA signaling and improves salt stress tolerance (Fang et al., 2017).

In our study, the expression levels of *PlMYB308* mirrored the effects of hormonal treatments during flower senescence. We used silencing and overexpression experiments to assess whether *PlMYB308* plays an essential role in flower senescence. Because a stable genetic transformation system of herbaceous peony has not been established, we used tobacco as a heterologous expression model system for studying the function of *PlMYB308*. The overexpression and silencing of *PlMYB308* reduced and increased flower longevity, respectively, which suggested that PlMYB308 plays an important role in the regulation of flower senescence. Our research showed that overexpression and silencing of *PlMYB308* influenced the production of ethylene, ABA, and GA in petals, suggesting that PlMYB308 positively regulates flower senescence by altering endogenous hormones contents.

We supposed that *PlMYB308* might be involved in the regulation of flower senescence through its interaction with endogenous hormone biosynthesis pathways. The biosynthesis pathways of various hormones have been extensively studied in plants. In GA biosynthesis, GA20-oxidase and GA3-oxidase are two important enzymes catalyzing the steps of bioactive GA production (Spray et al., 1996). Zeaxanthin epoxidase (ZEP), 9-*cis*-epoxycarotenoid dioxygenase (NCED), and aldehyde oxidase (AAO) are indispensable mediators of ABA biosynthesis in higher plants. The enzyme 1-aminocyclopropane-1-carboxylate synthase (ACS), which catalyzes the conversion of *S*-adenosylmethionine to ACC, as well as ACC oxidase (ACO), which converts ACC into ethylene, are two key enzymes required for ethylene synthesis (Zarembinski and Theologis, 1994). We detected the transcript abundances of 12 hormone biosynthetic genes and found that the transcript levels of *GA3ox1*, *ACO1*, *ACO3*, *ACS1*, and *NCED2* were variable in *PlMYB308*-silenced and *PlMYB308*-overexpressing plants. A dual-luciferase assay revealed the direct binding of PlMYB308 to the promoter of *PlACO1*. As we found significant differences between 62-SK-empty + *ProPlACO3* and 62-SK-PlMYB308 + *ProPlACO3*, as well as 62-SK-empty + *ProPlACS1* and 62-SK-PlMYB308 + *ProPlACS1*, at the level of 0.01 < *p* < 0.05 tested by Student’s *t*-test, we supposed that the weak interaction with *PlACO3* or *PlACS1* to PlMYB308 may be a false positive result. Furthermore, we used a yeast one-hybrid assay to verify the results of the dual-luciferase assay. The results of the yeast one-hybrid assay confirmed that PlMYB308 does indeed bind to the *PlACO1* promoter but could not bind to *PlACO3* and *PlACS1* promoters. Therefore, we demonstrated that PlMYB308 is involved in the regulation of flower senescence by targeting the ethylene biosynthesis pathway by activating *PlACO1* expression.

The change in transcript levels is one of the most important regulatory modes across different organisms. The transcription of eukaryotic genes is mainly regulated by the interaction between *cis*-acting elements and *trans*-acting factors. The predictive analysis of the *PlACO1* promoter sequence showed five *cis*-acting elements, comprising a Box4, G-Box, GT1-motif, MYB, and TCT-motif. Among them, the Box4, G-Box, GT1-motif, and TCT-motif are conserved DNA modules involved in light responsiveness. Besides, G-Box is a 6-bp CACGAC *cis*-acting element that exists widely in the promoter regions responsive to ABA, jasmonic acid, ethylene, elicifor, and hypoxia signals (Shen and Zhang, 1996). In addition, tobacco MYB305 was shown by an electrophoretic mobility shift assay to bind to the G-Box element in the *NtACO1* promoter (Sablowski et al., 1995). In *Hevea brasiliensis*, HbMYB15, HbMYB21, HbMYB35, and HbMYB75 showed an interaction with the same 6-bp in an *in vitro* promoter-binding test. These reports showed that MYB proteins specifically bind to G-Box motifs, which is in accordance with our findings that PlMYB308 may directly target the G-Box-containing promoter of *PlACO1.*

### PlMYB308 May Participate in a Combinatorial Interaction Between Ethylene, Gibberellin, and Abscisic Acid During Flower Senescence

Ethylene, ABA, and GA are all involved in plant growth and development processes such as stomata formation, seed germination, and flower senescence (Woltering et al., 1996; Richards et al., 2001; Fleet and Sun, 2005), and the crosstalk between these three hormones has been demonstrated in many plants. Both ethylene and ABA play important roles in the senescence of cut flowers, and they can promote the synthesis of one another (Doorn and Woltering, 2008). In *Oryza sativa*, an ethylene-responsive factor, *OsDERF2*, plays a vital role in response to ABA signaling (Guo et al., 2017). In peony, ethylene and ABA both regulate the senescence process of cut flowers, and Wang et al., supposed that ABA may influence peony senescence by increasing its ethylene production (Wang and Dong, 2012). In this study, we showed that the expression of *PlMYB308* was up-regulated under ethylene and ABA treatment, suggesting that *PlMYB308* may play a role in the interaction between ethylene and ABA during senescence. Silencing *PlMYB308* down-regulated ethylene and ABA biosynthesis genes, and reduced ethylene and ABA content, while the over-expression of *PlMYB308* up-regulated them. As we discussed before, PlMYB308 is involved in flower senescence by promoting *PlACO1* expression in herbaceous peonies. We supposed that during herbaceous peony senescence, a high expression of *PlMYB308* initially accelerated ethylene biosynthesis, followed by a promotion of ABA accumulation, and then, these two hormones collaboratively resulted in flower senescence.

There is an antagonistic action between ethylene and GA. Active ethylene signaling results in decreased GA production. Previous evidence suggests that the reduction of GA levels enhances ethylene-mediated flower senescence in roses. *RhHB1* mediates the antagonistic effect of GA and ethylene during flower senescence (Lü et al., 2014). *RhNF-YC9*, an ethylene-inhibited TF, regulates the size of petal cells by mediating GA synthesis (Chen et al., 2020). In peony, Shi et al. (2008) found that flower senescence is always accompanied by an increase in ethylene levels and a decrease in GA levels. In our study, GA_3_ treatment remarkably delayed petal senescence and played an antagonistic role in ABA- and ethylene-induced petal senescence. This suggests that in herbaceous peony, GA also plays the antagonistic action of ABA and ethylene. *PlMYB308*-overexpressing transgenic tobacco plants showed a GA production lower than that in WT plants, which caused the repressed growth phenotypes of transgenic tobacco plants, such as shorter stems and smaller leaves, and shorter flower longevity. By contrast, when silencing *PlMYB308* in herbaceous peony petals, GA levels increased and senescence delayed. According to these results, we suppose that with the development of herbaceous peony flowers, the expression level of *PlMYB308* increases, the high expression level of *PlMYB308* accelerates ethylene accumulation, and the accumulation of ethylene reduces GA production, after which flower senescence occurs. We have drawn a model (Figure 7) to suggest that PlMYB308 may participate in a complex reciprocal interplay between ethylene, ABA, and GA, and ethylene may be the most important growth regulator during flower senescence in cut herbaceous peony. However, without *PlACO1*, we found no exact structural genes associated with ABA or GA biosynthesis binding to PlMYB308 in our work. Future work comprehensively analyzing the expression levels of expanded ABA and GA biosynthetic genes in *PlMYB308*-silenced and -overexpressing plants in warranted.

## Data Availability Statement

The datasets presented in this study can be found in online repositories. The names of the repository/repositories and accession number(s) can be found in the article/Supplementary Material.

## Author Contributions

DS, LN, and XJ designed the experiments. XJ, ZX, and MW performed the experiments. KW contributed to materials. XJ and DS conceived the research and analyzed the data. XJ wrote the manuscript. DS commented and approved the manuscript. LN conceived and coordinated the project. All authors contributed to the article and approved the submitted version.

## Conflict of Interest

The authors declare that the research was conducted in the absence of any commercial or financial relationships that could be construed as a potential conflict of interest.

## Publisher’s Note

All claims expressed in this article are solely those of the authors and do not necessarily represent those of their affiliated organizations, or those of the publisher, the editors and the reviewers. Any product that may be evaluated in this article, or claim that may be made by its manufacturer, is not guaranteed or endorsed by the publisher.

## Footnotes

1
http://smart.embl-heidelberg.de/smart/set_mode.cgi?NORMAL=1

2
https://www.ncbi.nlm.nih.gov/gene
