# Supplementary material for: PlMYB308 Regulates Flower Senescence by Modulating Ethylene Biosynthesis in Herbaceous Peony
Source: Front Plant Sci. 2022 May 31;13:872442. doi: 10.3389/fpls.2022.872442 (PMC9194951; doi:10.3389/fpls.2022.872442)
Supplement: Supplementary file 1 [file Data_Sheet_1.docx]

**Fig. S1.**


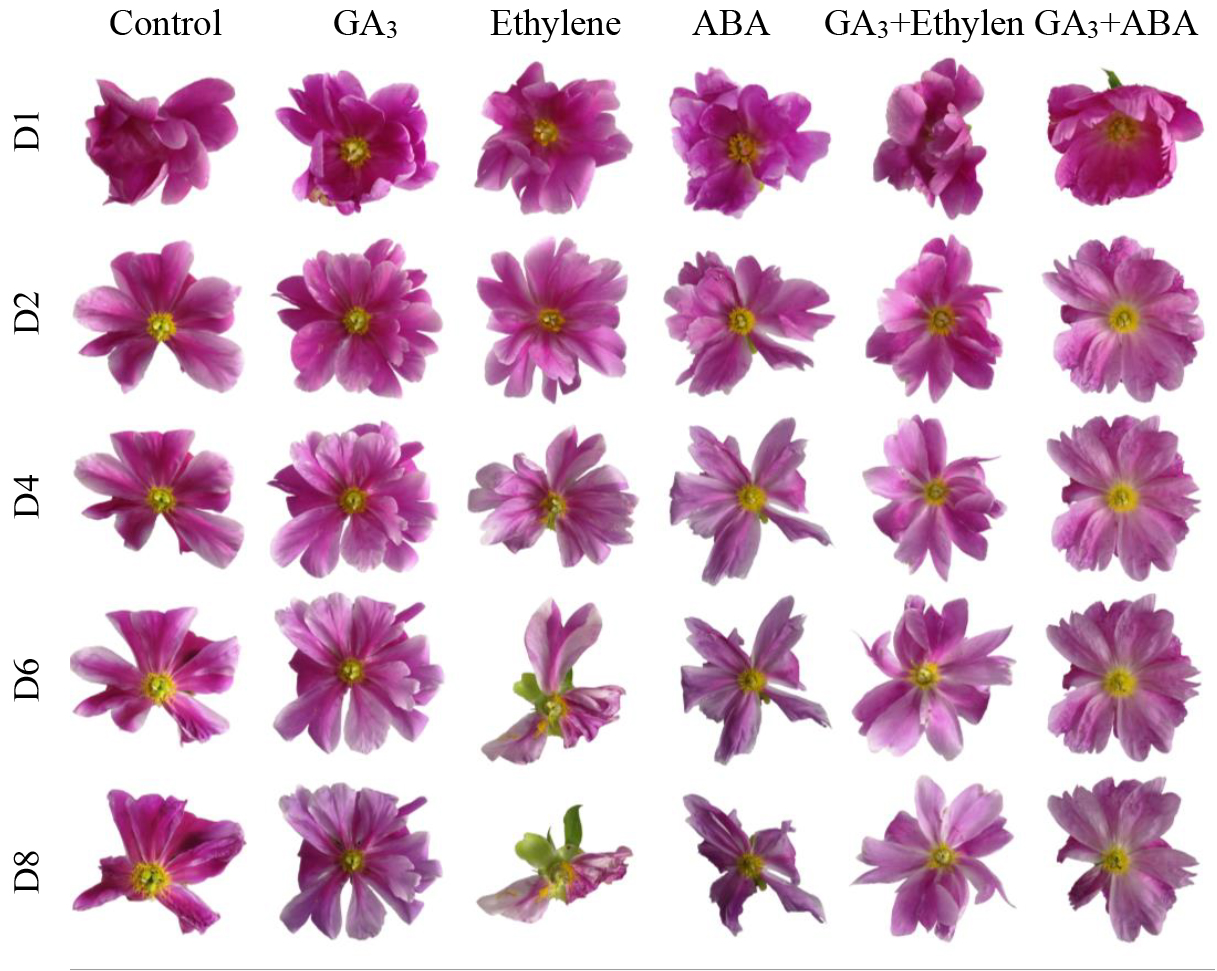


Senescence process of herbaceous peony flowers treated with various hormones. After 24h treatment with distilled water, 50μM GA_3_, 200μM ethephon, 100μM ABA, combinations of 50μM GA_3_ and 200μM ethephon and combinations of 50μM GA_3_ and 100μM ABA respectively, the flowers were kept in distilled water and the photos were taken at first day (D1), second day (D2), fourth day (D4), sixth day (D6) and eighth day (D8) after treatment.

**Fig. S2.**
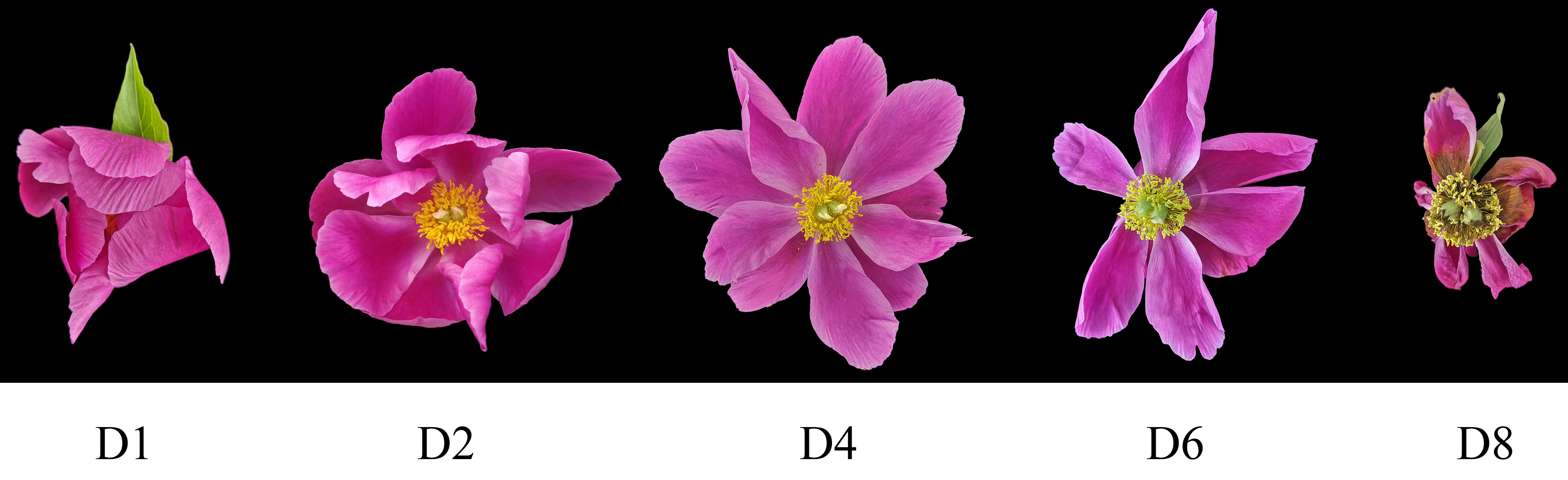


Different stages of herbaceous peony flower senescence.

**
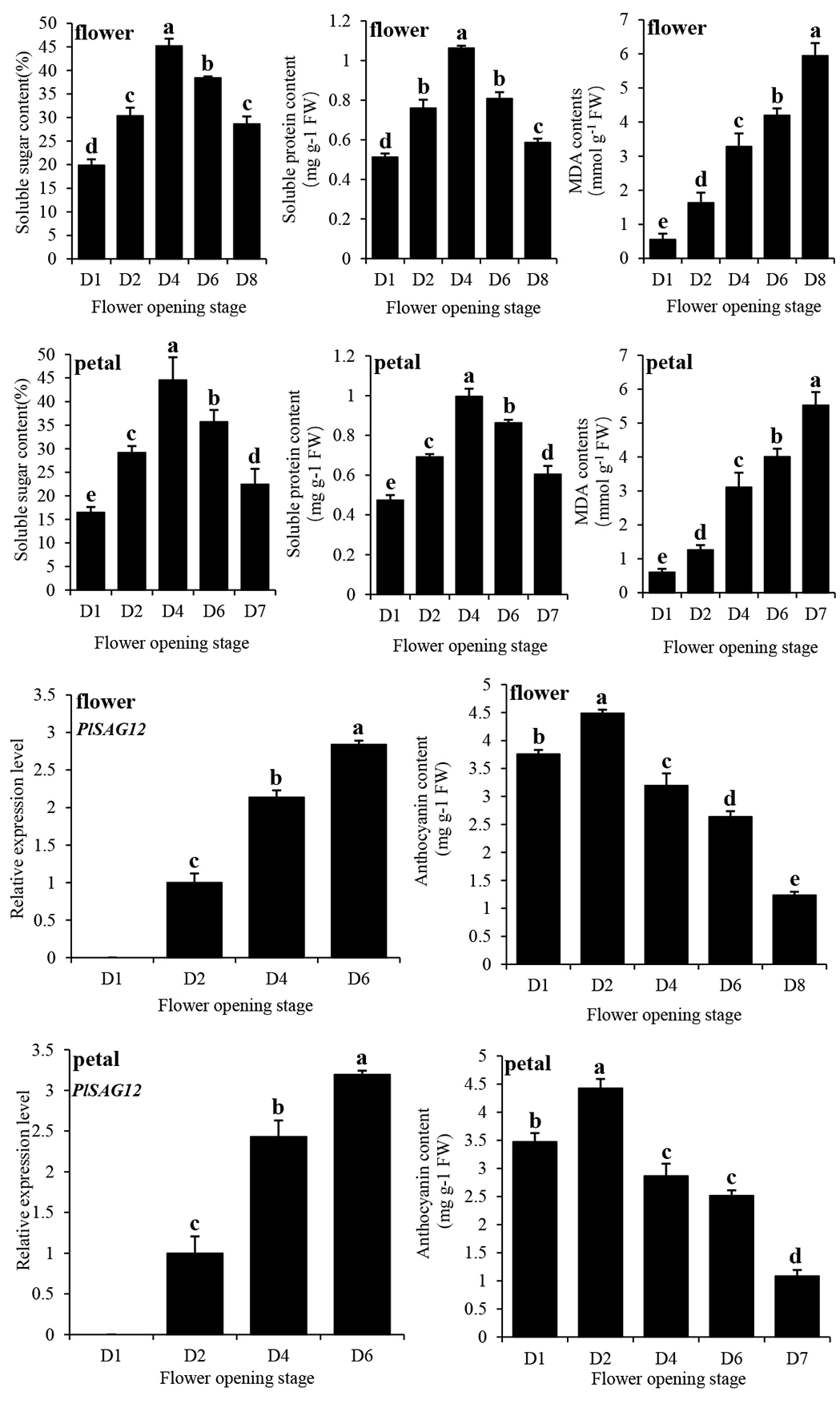
Fig. S3.**

Senescence related markers trends during flower senescence in petal discs and whole flowers.

**Fig. S4.**


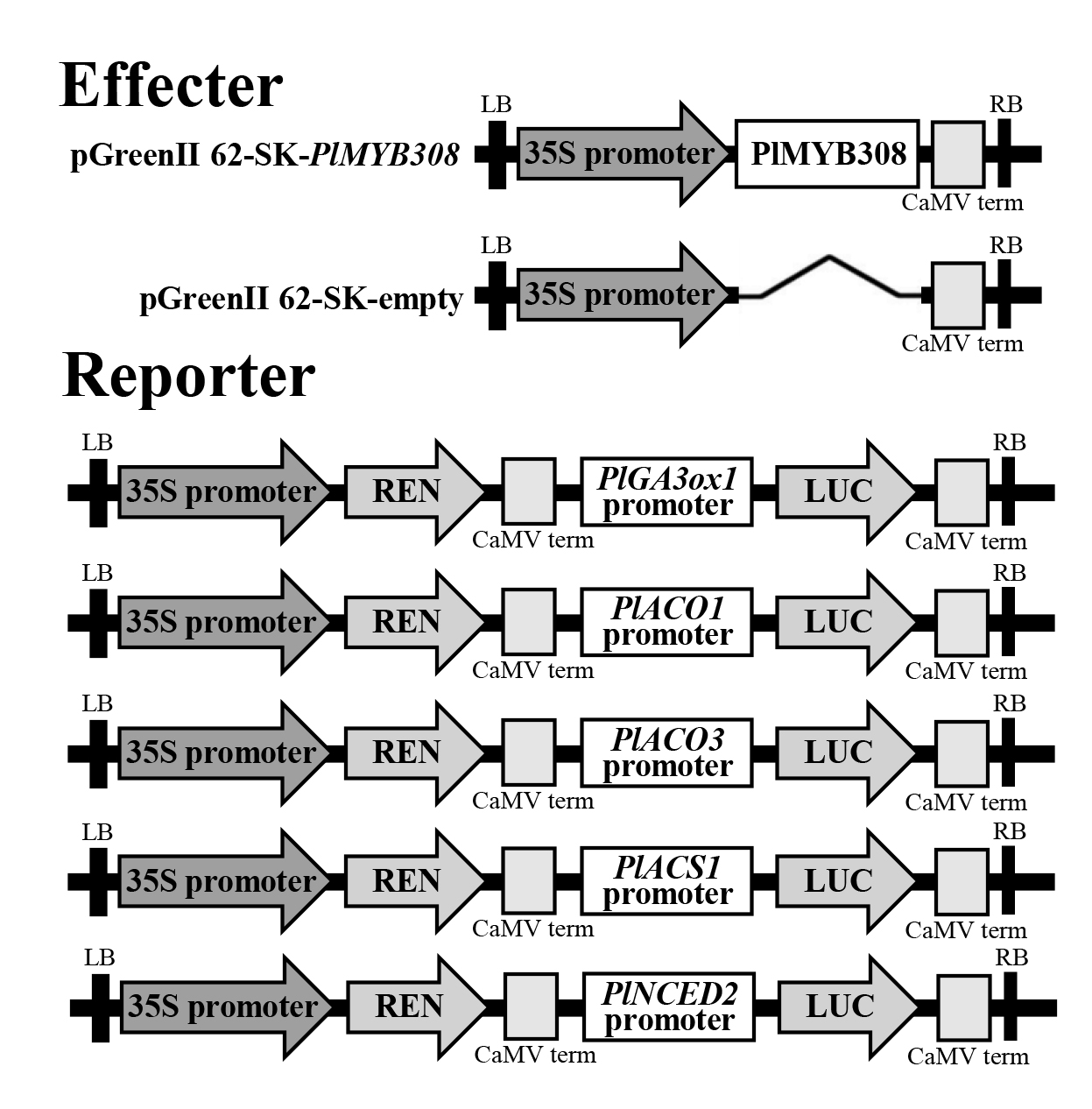


Schematic diagrams of the reporter and effector constructs used for the dual-luciferase assay.

**Table S1*.*** ***Cis*-acting component analysis in the promoter of *PlGA3ox1.***

| **Site Name** | **Position** | **sequence** | **function** |
| --- | --- | --- | --- |
| ABRE | 439 | AACCCGG | cis-acting element involved in the abscisic acid responsiveness |
| Box 4 | 72 | ATTAAT | part of a conserved DNA module involved in light responsiveness |
| CGTCA-motif | 647 | CGTCA | cis-acting regulatory element involved in the MeJA-responsiveness |
| CGTCA-motif | 967 | CGTCA | cis-acting regulatory element involved in the MeJA-responsiveness |
| GT1-motif | 807 | GGTTAAT | light responsive element |
| P-box | 236 | CCTTTTG | gibberellin-responsive element |
| TGACG-motif | 270 | TGACG | cis-acting regulatory element involved in the MeJA-responsiveness |
| TGACG-motif | 53 | TGACG | cis-acting regulatory element involved in the MeJA-responsiveness |

**Table S2*. Cis*-acting component analysis in the promoter of *PlACO1.***

| **Site Name** | **Position** | **sequence** | **function** |
| --- | --- | --- | --- |
| Box 4 | 327 | ATTAAT | part of a conserved DNA module involved in light responsiveness |
| Box 4 | 457 | ATTAAT | part of a conserved DNA module involved in light responsiveness |
| G-box | 849 | CACGAC | cis-acting regulatory element involved in light responsiveness |
| GT1-motif | 416 | GGTTAA | light responsive element |
| MYB | 19 | CAACCA | unknow |
| MYB | 827 | CAACCA | unknow |
| TCT-motif | 225 | TCTTAC | part of a light responsive element |
| TCT-motif | 488 | TCTTAC | part of a light responsive element |

**Table S3*. Cis*-acting component analysis in the promoter of *PlACO3.***

| **Site Name** | **Position** | **sequence** | **function** |
| --- | --- | --- | --- |
| MYB | 412 | CAACAG | unknow |
| TCT-motif | 78 | TCTTAC | part of a light responsive element |

**Table S4*.*** ***Cis*-acting component analysis in the promoter of *PlACS1.***

| **Site Name** | **Position** | **sequence** | **function** |
| --- | --- | --- | --- |
| ABRE | 1382 | ACGTG | cis-acting element involved in the abscisic acid responsiveness |
| ABRE | 1399 | ACGTG | cis-acting element involved in the abscisic acid responsiveness |
| Box 4 | 537 | ATTAAT | part of a conserved DNA module involved in light responsiveness |
| CAT-box | 66 | GCCACT | cis-acting regulatory element related to meristem expression |
| CGTCA-motif | 287 | CGTCA | cis-acting regulatory element involved in the MeJA-responsiveness |
| CGTCA-motif | 341 | CGTCA | cis-acting regulatory element involved in the MeJA-responsiveness |
| CGTCA-motif | 308 | CGTCA | cis-acting regulatory element involved in the MeJA-responsiveness |
| CGTCA-motif | 362 | CGTCA | cis-acting regulatory element involved in the MeJA-responsiveness |
| G-Box | 469 | CACGTT | cis-acting regulatory element involved in light responsiveness |
| G-box | 1398 | TACGTG | cis-acting regulatory element involved in light responsiveness |
| G-box | 339 | CACGTC | cis-acting regulatory element involved in light responsiveness |
| G-box | 653 | CACGAC | cis-acting regulatory element involved in light responsiveness |
| GARE-motif | 1293 | TCTGTTG | gibberellin-responsive element |
| GATA-motif | 1032 | AAGGATAATG | part of a light responsive element |
| LTR | 1064 | CCGAAA | cis-acting element involved in low-temperature responsiveness |
| MRE | 977 | AACCTAA | MYB binding site involved in light responsiveness |
| MYB recognition site | 226 | CCGTTG | unknow |
| MYB recognition site | 906 | CCGTTG | unknow |
| TGACG-motif | 446 | TGACG | cis-acting regulatory element involved in the MeJA-responsiveness |
| TGACG-motif | 572 | TGACG | cis-acting regulatory element involved in the MeJA-responsiveness |

**Table S5*. Cis*-acting component analysis in the promoter of *PlNCED2.***

| **Site Name** | **Position** | **sequence** | **function** |
| --- | --- | --- | --- |
| CGTCA-motif | 966 | CGTCA | cis-acting regulatory element involved in the MeJA-responsiveness |
| GT1-motif | 549 | GGTTAA | light responsive element |
| MYB | 864 | CAACCA | unknow |
| MYB | 1148 | CAACAG | unknow |
| Sp1 | 1036 | GGGCGG | light responsive element |
| TGACG-motif | 963 | TGACG | cis-acting regulatory element involved in the MeJA-responsiveness |
| TGACG-motif | 1120 | TGACG | cis-acting regulatory element involved in the MeJA-responsiveness |

CGTCA-motif

TGACG-motif

Sp1

MYB

MYB

**Table S6.** **Genes specific primers used for gene isolation and expression analysis.**

| Primer Name | Primer Sequences | Usage |
| --- | --- | --- |
| *PlMYB308*-F | ATGGTACCATGGGAAGGTCTCCTTGTTG | Gene isolation |
| *PlMYB308*-R | ATGTCGACTTATTTCATCTCCAATCTTC |  |
| *vigsPlMYB308*-F | GAATTAGTCCCCCTTATCAACAC | Gene isolation |
| *vigsPlMYB308*-R | ATCTTCTGTAATCCAAAGAAGCA |  |
| *PlMYB308*-F | TCCTGACCTGAACCTTGAACTTA | qRT-PCR |
| *PlMYB308*-R | TAAGAAATCATAACCCGTGTTGC |  |
| *PlGA2ox1*-F | GCCTGCATTTCGTACTATTTGTC | qRT-PCR |
| *PlGA2ox1*-R | GCTCCTGTTAAGAATGTGATTGG |  |
| *PlGA2ox2*-F | ATCGTTGGATCTCAAGAGAGTGA | qRT-PCR |
| *PlGA2ox2*-R | TTCAGTGTTCAGTAGGCTCATCA |  |
| *PlGA20ox1*-F | CCTGGGATTGTTATTAGTGTCCA | qRT-PCR |
| *PlGA20ox1*-R | GGCGTTCCATTACTCCTAATTTC |  |
| *PlGA3ox1*-F | TGGTGGAGGATGGTAAAGAGTAA | qRT-PCR |
| *PlGA3ox1*-R | CTAGCAGAACGAGTGTGGCTATT |  |
| *PlACO1*-F | AATACCCTCAATGTCCTCAACCT | qRT-PCR |
| *PlACO1*-R | CTACCGTTCTTGTCTGCCATAAC |  |
| *PlACO3*-F | TTCCATGTTTACTCCAAGAAAGG | qRT-PCR |
| *PlACO3*-R | AAAGCCATTGAAATACCATCCTC |  |
| *PlACS1*-F | AAACGACATGGATGGTGTTAATC | qRT-PCR |
| *PlACS1*-R | TTTGAGTTGAAACTAGCCCAAAG |  |
| *PlACS7*-F | CTAAAACCTGGAAAGCCAAGAT | qRT-PCR |
| *PlACS7*-R | GCAAAAACATTCATCTTGTCTC |  |
| *PlZEP*-F | TACAAGGCATATTTGGGTGTAGG | qRT-PCR |
| *PlZEP*-R | CTAGGTGGTCTCCCTTCAAGATT |  |
| *PlAAO*-F | TACCCAAGGACTGAAACGATAGA | qRT-PCR |
| *PlAAO*-R | CAGCCATCTGTTTAACCTTTGTC |  |
| *PlNCED1*-F | ATTTTCCCCCCCTACACTCTCA | qRT-PCR |
| *PlNCED1*-R | AAGAAGAAGAAGACTGTTTGGG |  |
| *PlNCED2*-F | TGTCTCTAAGCATCCCTCTGGTA | qRT-PCR |
| *PlNCED2*-R | TAAAGTTGGAAGCCTCTGTCAAG |  |
| *NtGA2ox1*-F | TGGTGGTCTTAACTAAACCTGG | qRT-PCR |
| *NtGA2ox1*-R | GACGCTATGGTTAATGACTTTG |  |
| *NtGA2ox2*-F | TCAGCACTTCTCCAAAGACAAC | qRT-PCR |
| *NtGA2ox2*-R | GGGAGAGGAGAAGAATTTAATG |  |
| *NtGA20ox2*-F | GCATTTCTCAATGAACACCTTTC | qRT-PCR |
| *NtGA20ox2*-R | GTTGTTGTGTTGCCACTGAATTA |  |
| *NtGA3ox1*-F | TTCTAAATTGGTAGATCAAGGG | qRT-PCR |
| *NtGA3ox1*-R | ACTACTAGCCAAATTAGCCAAC |  |
|  |  |  |
| *NtACO1*-F | GCCAAGTACCTTCTTTCCTTCAT | qRT-PCR |
| *NtACO1*-R | TCACATTGTCCATCAGGTTAGTG |  |
| *NtACO3*-F | AATGGTTGCTCAGAGTTTCATGT | qRT-PCR |
| *NtACO3*-R | GACCTTGAAAAACTGGTCTTCCT |  |
| *NtACS1*-F | CACAACTGAAGGAGCTGAAGATT | qRT-PCR |
| *NtACS1*-R | CTCCACTCATTACTACGCGACTT |  |
| *NtACS7*-F | TTATTCTTGCTGATCCTGGTGAT | qRT-PCR |
| *NtACS7*-R | TCATAGGCTGATTCTAAGGCTTG |  |
| *NtZEP*-F | ATGGCATCAACTTACAAGGCTTA | qRT-PCR |
| *NtZEP*-R | CTCAATTGGTCATTTGCTTTCTC |  |
| *NtAAO*-F | TTAACTGATCATTCCCCAGCTTA | qRT-PCR |
| *NtAAO*-R | AACAAGTCTCAGAACGCAAGAAC |  |
| *NtNCED1*-F | CTACAACTCCTGCTTCAAATAC | qRT-PCR |
| *NtNCED1*-R | GGAATAGTAGTAGTTTTGGGTG |  |
| *NtNCED2*-F | TTTTCCCTCCCTACAAAATCAAT | qRT-PCR |
| *NtNCED2*-R | GGAAAAGATTCCAGTCAGGAGTT |  |
